# Supplementary material for: Promoter nucleosome dynamics regulated by signalling through the CTD code
Source: eLife. 2015 Jun 22;4:e09008. doi: 10.7554/eLife.09008 (PMC4502402; doi:10.7554/eLife.09008)
Supplement: Supplementary file 1. — Oligonucleotides used in this study. DOI: http://dx.doi.org/10.7554/eLife.09008.020 [file elife09008s003.docx]

**Supplementary file 1**

**Oligonucleotides used in this study**

*ste11* locus:

Amplicon -1: located 2363 bp upstream of the *ste11* ATG

1077: gtgacaaatgctttcgcatc

1078: ggcgtaatacgcgatgttac

Amplicon 1: located 2211 bp upstream of the *ste11* ATG

1017: gttggttgccaatgcgtatg

1018: tacgacgcgaaacaattcaacg

Amplicon 2: located 1377 bp upstream of the *ste11* ATG

1015: ctttgtctctaccaccatagttatcc

1016: ccaagcgatagaataacgatgcg

Amplicon 3: located 121 bp downstream of the *ste11* ATG

968: tcgtcgagatcgccaagc

969: ggcactttcatttctccacagc

Note that this amplicon was also used for the quantification of the mRNA in Q-RT-PCR

Amplicon 4: located 1114 bp downstream of the *ste11* ATG

970: gccatacttcttacccagcaatg

971: cagaattggtaacactagacgaagag

*adh1* locus:

Amplicon 1: located 132 bp upstream of the *adh1* ATG

819: ttttgctttgccgatgttactt

820: cacctgtccaccacccac

Amplicon used for the quantification of the mRNA in Q-RT-PCR, located 309 bp downstream of the *adh1* ATG:

783: attggtgaccgtgttggtg

784: gaaagttgaatgtgagggcag

*sam1* locus:

Amplicon used for the quantification of the mRNA in Q-RT-PCR, located 987 bp downstream of the *sam1* ATG:

986: ccgctgccttgtccaactc

987: ggaagaagtgccgtaagtattgac

*act1* locus:

Amplicon used for the normalization of the Q-RT-PCR, located 948 bp downstream of the *act1* ATG:

739: ccactatgtatcccggtattgc

740: caatcttgaccttcatggagct

*inv1* locus:

Amplicon used for the synthesis of the multiprimed labeled probe used in northern blotting:

1939: ttgctgagcgtcaggctattg

2035: atctgtccaggtgttgctcc

Amplicon -1: located 1592 bp upstream of the *inv1* ATG

1962: gcaccaccattctgtaaaactc

1963: ggggatgtgttcttatttgcg

Amplicon 1: located 1072 bp upstream of the *inv1* ATG

1960: cttagacattgtcagcacaacc

1961: aagtcgatcatgaccttccg

Amplicon 2: located 632 bp downstream of the *inv1* ATG

1939: ttgctgagcgtcaggctattg

1940: ggtcgcggaattgaagtg

*fbp1* locus:

Amplicon 1: located 276 bp upstream of the *fbp1* ATG

2163: cgcttccatcatggctc

2164: tgtgttgtctctctccagtg

Amplicon 2: located 231 bp downstream of the *fbp1* ATG

1427: caattcagcttcaaattcatcgc

1428: cttttgttcatcgccagtgg

Amplicon 3: located 1114 bp downstream of the *fbp1* ATG

2165: tagttctttgaagtcctctccg

2166: ggagatccatgtttatgatgatgc

*cam1* locus:

Amplicon used for the synthesis of the multiprimed labeled probe used in northern blotting:

2167: cccgtaaccttacagatgag

2168: aaatgacacgagagaattcttcg

*IME1* locus:

Amplicon 1: located 323 bp upstream of the *IME1* ATG

2009: tgatcttgttccttgcctgttg

2010: aataccgaactacaagggaagc

Amplicon used for the quantification of the mRNA in Q-RT-PCR, located 385 bp downstream of the *IME1* ATG:

1937: ctacgaaggactgcacaatgg

1938: ttgaatgatgagtggaacgtagatg

*ACT1* locus:

Amplicon used for the normalization of the Q-RT-PCR, located 948 bp downstream of the *ACT1* ATG:

1984: gtgatgtcgatgtccgtaagg

1985: tcatggaagatggagccaaag

Oligonucleotides used for nucleosome scanning (**Figure 2 – Figure Supplement 1, Figure 3**)

A F aactgccggcacttgttttc

AR cggcaatttgcgaaaccg

BF gcttaaaccttacctataataccctgc

BR tggagagttaaggaggggtg

CF tgctttctccacccctcc

CR gcaaggcaaaatgacaagaagc

DF cattggaagactagtcgttggc

DR gattagtgtggcacttggca

EF acgaaaagtagctttgcgagac

ER cttgcctacaaacgatgtaacaca

FF tccctgactttgtttgctgtg

FR ggggaagggacaaaattcaca

GF ctgtgaattttgtcccttcccc

GR ggcgtaatacgcgatgttactg

HF attgtcagtaacatcgcgtattacg

HR ctcactatgtaagtcaccaggtatgt

IF acatacctggtgacttacatagtgag

IR caggtttaagattgaaaagtcagaatgc

JF acaaagcattattagcattctgacttttc

JR cgaggcaaaagctctcaaagaa

KF gcattttatttttctttgagagcttttgc

KR gctgcaatcaagacaaagacc

LF caacttaacttaacttttctggtctttg

LR gctaagccaaagcaaaccaag

MF ccattcttatttcactcccgttc

MR gcatagcgaatgtgtgaaaaca

NF gtgtggtttctttccttccg

NR gtagtttggggtgaacggaac

OF cgcctaattttcggtaaattgtagc

OR aaaaatacaggtactacgagcgg

PF tatttgtggtgcatgccatc

PR agtcattgccattggaattttgc

QF acttacctttccgacattcttgt

QR tcccttccatcatccctgg

**Strains used in this study**

94: *wt*

461: *lsk1::ura4 ura4-D18 leu1-32*

552: *rpb1 CTD S2A-kanR*

1025: *lsk1 ΔN*

528: *lsk1-TAP-kanR*

904: *sty1-TAP-kanR*

1122: *lsk1 12 SA-TAP-kanR*

920: *lsk1 4 SA-TAP-kanR*

949: *lsk1 4 SE-TAP-kanR*

594: *rpb3-HA-kanR*

1234: *rpb3-HA-kanR lsk1 12 SA-TAP-kanR*

1110: *lsk1-as*

1169: *gcn5-myc-natR*

1180: *gcn5-myc-natR lsk1::kanR*

1181: *h3.1-h4.1::kanR h3.2 K14R h3.3-h4.3::natR*

1146: *h3.1-h4.1::kanR h3.2 K36R h3.3-h4.3::natR*

1239: *h3.1-h4.1::kanR h3.2 K36Q h3.3-h4.3::natR*

1162: *h3.1-h4.1::kanR h3.3-h4.3::natR*

1598: *h3.1-h4.1::kanR h3.2 K4R h3.3-h4.3::natR*

1116: *clr3::natR*

1106: *hos2::hphR*

1196: *hst2::bleR*

1199: *hst4::natR*

1211: *sir2::kanR*

1126: *clr6-1*

1255: *hos2-TAP-kanR*

1274: *rpb1-CTD-Δ-mce1-natR*

926: *rpb1-CTD-Δ−-mce1-natR*

1223: *rpb1-CTD-Δ−-S2A-mce1-natR*

1294: *rpb1-CTD-Δ−-mce1-natR hos2-TAP-kanR*

938: *rpb1-CTD-Δ−2-natR*

1236: *set1-TAP*

1364: *lsk1::kanR hos2::hphR clr3::natR*

1372: *lsk1::kanR hos2::hphR*

979: *lsk1-TAP-kanR sty1::natR*

903: *sty1::kanR*

1050: *set2::kanR*

1271: *set1::natR*

1490: *png2::kanR*

1491: *png2::kanR lsk1::natR*

1558*: leo1::kanR*

0037: a/α

0040: a/α *ctk1::kanR* / *ctk1::natR*

0054: a/α *set3::natR* / *set3::natR*

0055: a/α *set3::natR* / *set3::bleR ctk1::kanR* / *ctk1::hphR*
